# Fibula allograft with cannulated screw fixation versus ordinary cannulated screw fixation for femoral neck fractures: a 10-year retrospective comparative study

**DOI:** 10.1186/s13018-023-04002-1

**Published:** 2023-08-05

**Authors:** Yangwenxiang Wei, Tianye Lin, Yuhao Liu, Zhenqiu Chen, Chi Zhou

**Affiliations:** 1 The First Clinical of Medical School, Guangzhou University of Chinese Medicine, No. 12 Jichang Road, Baiyun District, Guangzhou, 510405 Guangdong Province China; 2 The Third Affiliated Hospital, Guangzhou University of Chinese Medicine, Guangzhou, 510240 Guangdong China; 3 The Department of Orthopedics, The First Affiliated Hospital of Guangzhou University of Chinese Medicine, No. 16 Jichang Road, Baiyun District, Guangzhou, 510405 Guangdong Province China; 4 The Lab of Orthopaedics of Chinese Medicine of Lingnan Medical Research Center, Guangzhou University of Chinese Medicine, Guangzhou, 510405 Guangdong China

**Keywords:** Femoral neck fractures, Allograft of fibula, Cannulated screws, Therapeutic effect, Complications

## Abstract

**Background:**

For femoral neck fractures in young and middle-aged patients, both fibula allograft with cannulated screw fixation and ordinary cannulated screw fixation are clinically effective treatments. However, for unstable femoral neck fractures, ordinary cannulated screw fixation is characterized by a high risk of postoperative complications and a high rate of mechanical failure after internal fixation. For this study, we systematically compared the long-term efficacy and postoperative complications of these two procedures.

**Methods:**

A total of 156 subjects diagnosed as femoral neck fractures participated in our study. Subjects in the combination group underwent fibula allograft with cannulated screw fixation (*n* = 76), and those in the control group were treated with ordinary cannulated screw fixation (*n* = 80). Baseline characteristics, perioperative outcomes, Harris hip score (HHS) and EuroQoL five-dimension questionnaire (EQ-5D); and the incidence of postoperative and bone healing complications in the two groups were recorded and compared.

**Results:**

The average follow-up time was more than 10 years. Intra-operative blood loss significantly increased in the combination group compared with the control group (*P* < 0.05). There were significantly improved performances in healing time, the time course of recovery of full-weight-bearing stepping, HHS and EQ-5D scores in the combination group compared with the control group (*P* < 0.05). Besides, the incidence rates of femoral head necrosis, nonunion, femoral neck shortening and total hip replacement were significantly lower in the combination group than those in the control group (*P* < 0.05).

**Conclusion:**

Fibula allograft with cannulated screw fixation shows a better long-term therapeutic effect than ordinary cannulated screw fixation for femoral neck fractures in young and middle-aged patients. Patients receiving the combination strategy have faster and high-quality functional recovery after femoral neck fractures and a lower incidence rate of postoperative complications.

## Introduction

Femoral neck fractures are among the most common types of fractures, accounting for 3.6% of all fractures and 48–54% of hip fractures. Approximately 63 million will suffer from femoral neck fractures through 2050, about half of whom will be Asians [1]. China saw a growing population of femoral neck fractures over the past decade [2, 3], many of whom were experiencing great clinical and economic burdens. Hence the advantages of total hip arthroplasty may not be appealing [4]. For young patients who are more physically active, loosening, dislocation or infection of the prosthesis after hip replacement and multiple revisions for failed hip replacement are their major concerns [1]. Previous research has proved the feasibility of intramedullary fixation with cannulated screws to meet young patients with high functional needs, especially in somewhere where total hip arthroplasty is not easily accessible or high-cost [4, 5]. Therefore, the preservation of the femoral head especially for young patients is the most important factor to consider during treatment [6].

Recently, there has been a surge of research into safe and effective methods of internal fixation for femoral neck fractures, including cannulated screw fixation, proximal femoral locking plates, and sliding hip screw fixation with or without additional dislocating screws. However, the recognized best treatment is still elusive despite many useful strategies exist [7]. Previous studies reported that the incidence rates of osteonecrosis and nonunion were 14.3% and 9.3% after femoral neck fractures [8]. Currently, there is still no method to effectively prevent these incidents. Fibula allograft is a simple procedure that provides strong support for better treatment of early-stage ONFH with lower complications [9]. Our previous study reported a high rate of 97.5% for fibula allograft in early femoral head necrosis after a mean follow-up of 7 years [10]. Additionally, a further study found that fibula bone graft combined with cancellous screw fixation for neglected femoral neck fractures achieved good efficacy with fewer complications [11]. For these reasons, we hypothesized that fibula allograft with cannulated screw fixation would be superior to ordinary cannulated screw fixation with regard to hip function, living quality, and the rates of nonunion, femoral head necrosis and femoral neck shortening in patients with femoral neck fractures.

For this study, the purpose was to retrospectively analyze and compare the long-term clinical outcomes and postoperative and bone healing complications of fibula allograft combined with cannulated screw fixation versus ordinary cannulated screw fixation for the treatment of femoral neck fractures and to investigate "pros and cons" of this combination strategy.

## Materials and methods

### The inclusion and exclusion criteria

Inclusion criteria are as follows: (*i*) patients aged 18–60 years; (*ii*) unilateral displaced transcervical femoral neck fracture; (*iii*) patients were treated with allogeneic fibula combined with cannulated screw fixation or ordinary cannulated screw fixation; (*iv*) postoperative follow-up time of more than 5 years.

Exclusion criteria are as follows: (*i*) surgical area with poor skin conditions or skin diseases; (*ii*) severe osteoporosis; (*iii*) pathological fractures or old fractures (more than 14 days); (*iv*) comorbid rheumatoid arthritis, osteomyelitis, severe cardiovascular and cerebrovascular diseases; (*v*) incomplete clinical data.

### Subjects

**Fig. 1:**
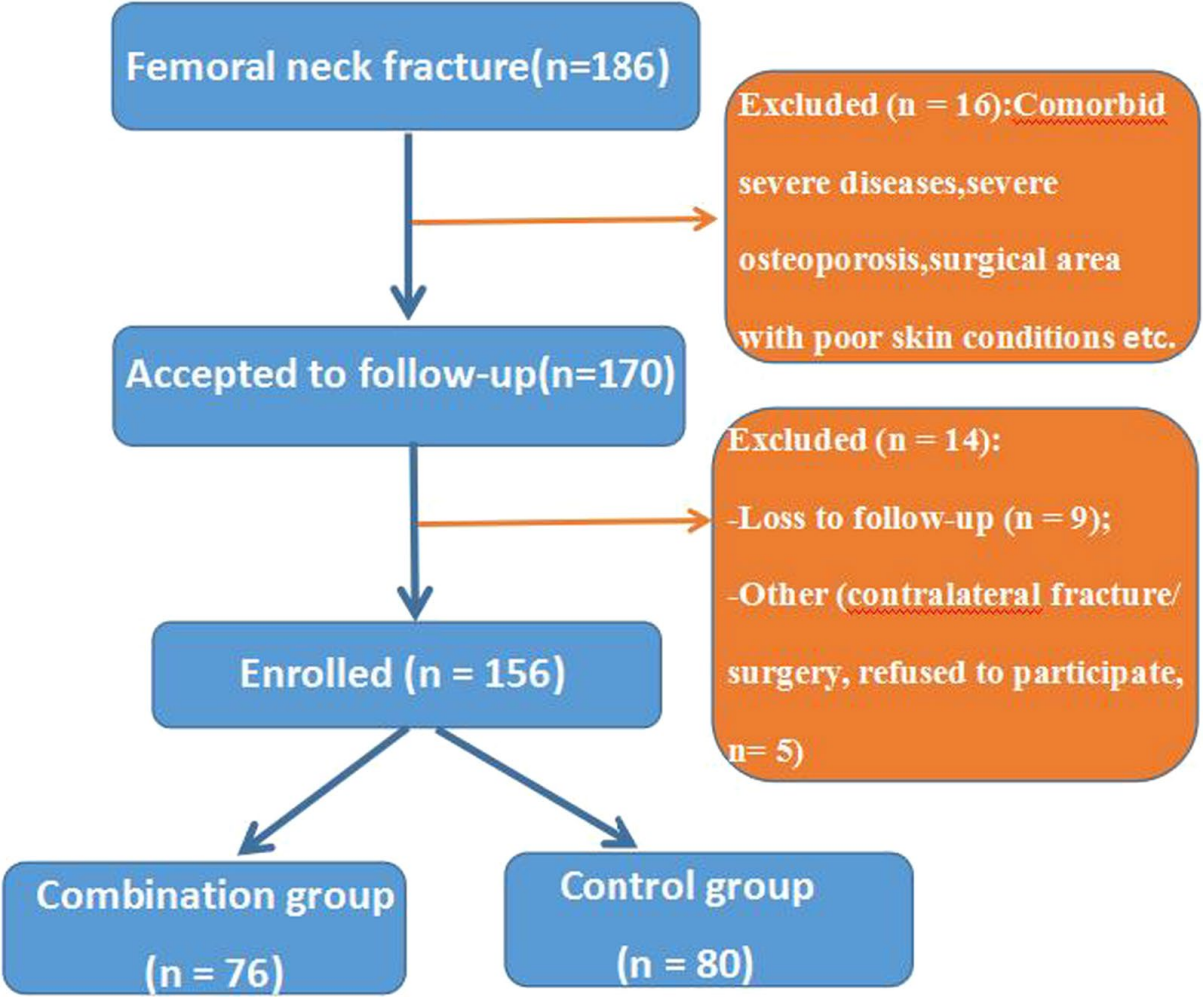
The eligibility flowchart of subjects in the combination and control groups

A total of 186 candidates who were diagnosed as femoral neck fractures at the First Affiliated Hospital of Guangzhou University of Traditional Chinese Medicine from January 2006 to January 2012 were initially involved in this study. Sixteen of them did not fulfill the inclusion criteria, 9 were lost to follow-up, and 5 withdrew from this study for other reasons (Fig. 1). The demographic characteristics and clinical profiles including age, gender, the mechanism of injury, the side of fracture, time to surgery, body mass index (BMI), and follow-up duration were recorded. Finally, 156 eligible subjects were selected and assigned to the combination group (fibula allograft combined with cannulated screw fixation, *n* = 76) and the control group (ordinary internal fixation with parallel cannulated screw in an inverted triangle configuration, *n* = 80) according to surgical protocols. All participants provided written informed consent, and the current study was approved by the Ethics Committee of the First Affiliated Hospital of Guangzhou University of Traditional Chinese Medicine(No: JK《2019》061).

### Surgical procedures

**Fig. 2:**
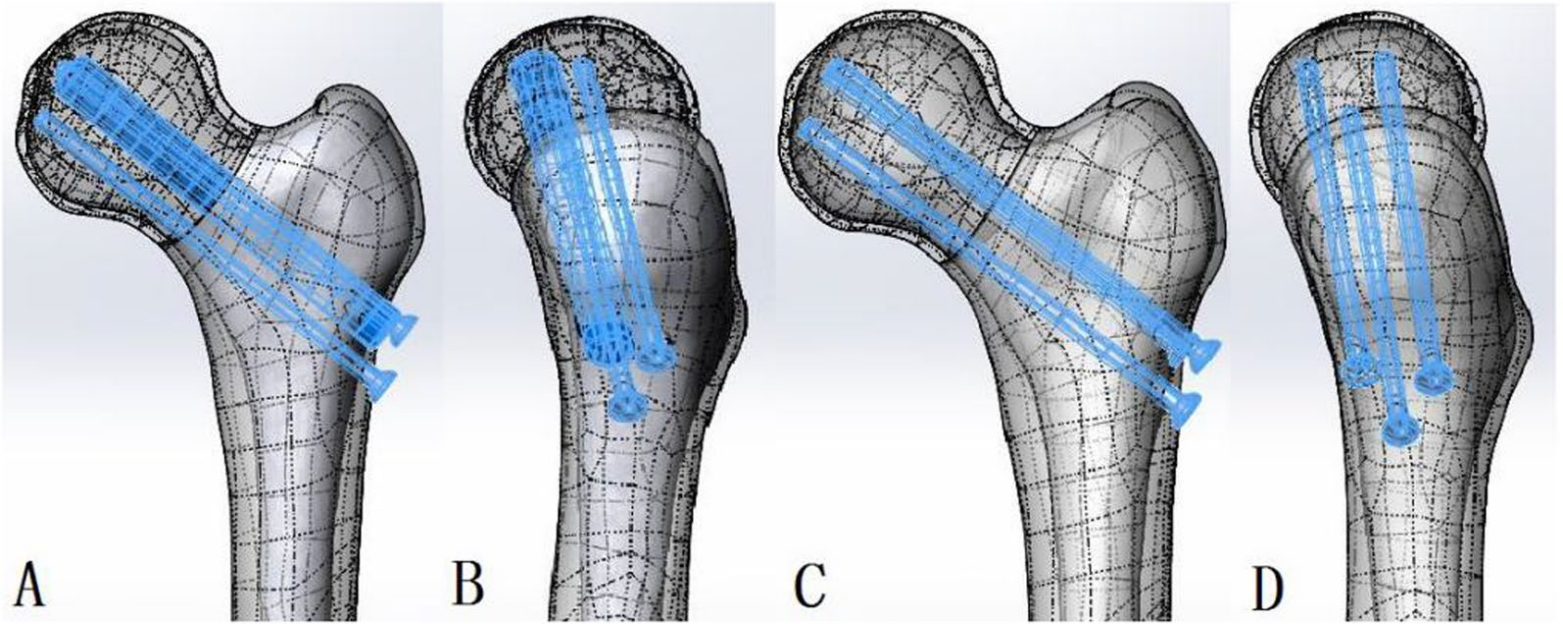
Surgical procedures for the combination group and the control group. **A** & **B**: fibula allograft with cannulated screw fixation (the combination group); **C** & **D**: ordinary internal fixation with parallel cannulated screw in an inverted triangle configuration (the control group)

Operations were performed by the same surgeon group. All subjects underwent lumbar anesthesia. The patient was placed supine with legs apart on an orthopedic table. A manual reduction for the fracture was performed, and the C arm X-ray guidance system was employed to confirm that the hip joint was in a good reduction position. The affected leg was slightly abducted and internally rotated. A longitudinal incision about 5 cm was made below the tip of the greater trochanter. One or two threaded Kirschner wires were inserted into the femoral head and neck to temporarily fix the fracture. The above procedures including drilling and insertion were to keep an anatomically reduced position and avoid inversion and recline. For subjects in the control group, three parallel guide needles were inserted into the femoral head along the longitudinal axis of femoral neck in an inverted triangle configuration. After confirming a proper screw placement, three 7.3-mm cannulated screws were screwed and finally pressed evenly, the distal threads completely passed the fracture line and the top of the screw reached 5–10 mm below the femoral head cartilage. Moreover, the screw should be as close to the cortex as possible [12]. Notably, to reduce stress concentration, the screw entry point should not be lower than the horizontal plane of the lesser trochanter. For subjects in the combination group, the first cannulated screw was placed within 3 mm from the anterior and inferior cortex of the femur. Subsequently, allogeneic fibula graft was inserted into the weight-bearing area of the anterior and upper femur. The top of the fibula graft should reach 5–8 mm below the femoral head cartilage. The diameter of the fibula graft was 11–12 mm. Because the bone tissue has a certain elasticity, the inner diameter of the implanted fibula column should be about 0.5–1 mm smaller than the allogeneic fibula column so that the fibula column could be implanted into the weight-bearing position by compression. The second cannulated screw were placed just below the allofibular column and tightly fixated. Notably, the top of the cannulated screw inside and below the fibula should not exceed the distal end of the fibula, and the two screws alongside the grafted fibular column formed a powerful mechanical construct (Fig. 2).

### Postoperative management

**Fig. 3:**
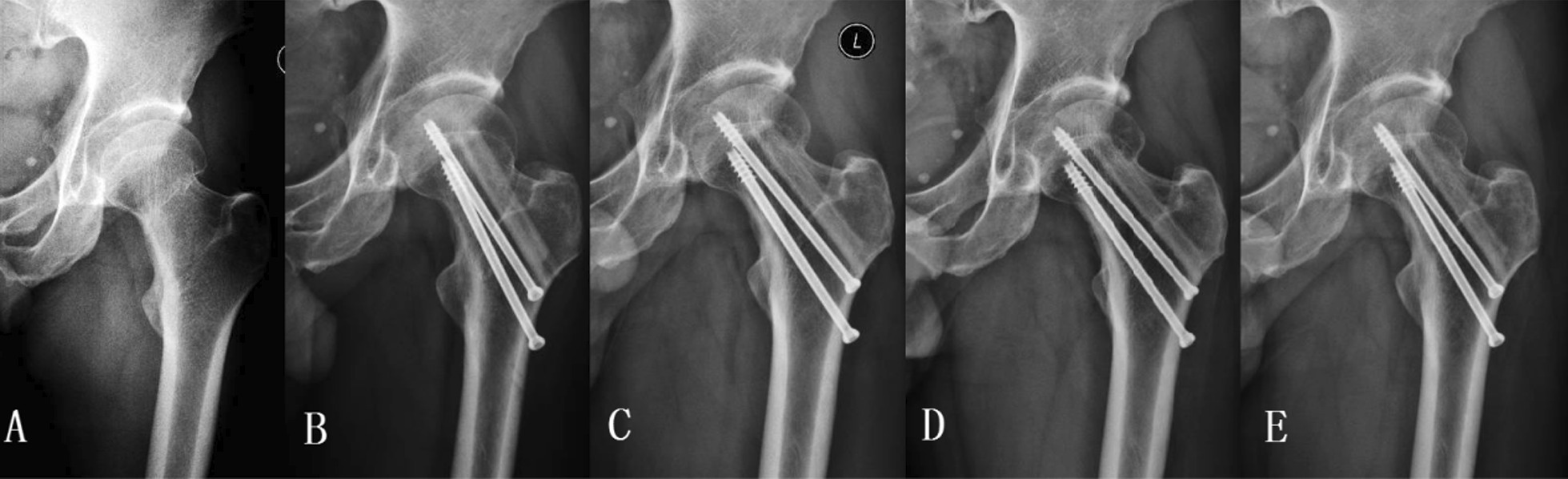
The radiograph illustrated fracture healing process in a 57-year-old man receiving fibula allograft combined with cannulated screw fixation. The anteroposterior X-ray view of the proximal femur at pre-operation (**A**), 3 months (**B**), and 1- (**C**), 2- (**D**) and 4-year (**E**) follow-up visits after operation. Femoral neck fracture healed well without complications such as femoral head necrosis

**Fig. 4:**
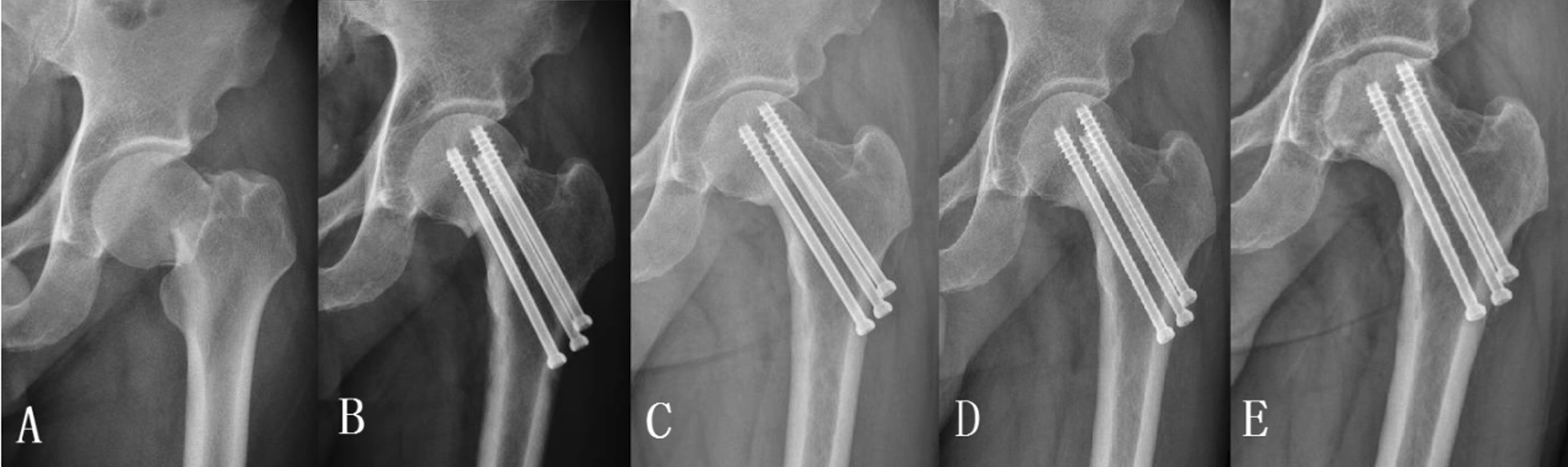
A 52-year-old man with left femoral neck fracture received ordinary cannulated screw fixation. The anteroposterior X-ray view of the proximal femur at pre-operation (**A**), 3 months (**B**), 9 months (**C**), and 2-(**D**) and 4-year (**E**) follow-up visits after operation. The left femoral neck fracture completely healed, but femoral head necrosis occurred

To avoid postoperative infections, antibiotics were carefully used according to an individual’s condition during the perioperative period. All subjects were administered by the subcutaneous injection of low-molecular-weight heparin to prevent venous thrombosis after surgery. Postoperative rehabilitation started from nonweight-bearing bilaterally in the first 6 weeks to partial weight-bearing in the following 6 weeks with crutches, and to full weight-bearing with or without crutches according to different phases of fracture healing. Each subject was followed up clinically and radiologically at 3, 6, and 12 months and every year postoperatively. At each follow-up visit, AP plain radiographs, computed tomography (CT) and magnetic resonance imaging (MRI) of the affected hip joint were reexamined (Figs. 3 and 4).

### Measurements

Medical records consisted of operative time, intra-operative blood loss, incidence of postoperative and bone healing complications, fracture healing time, femoral neck shortening and the time course of recovery of full-weight-bearing. The HHS was used to evaluate postoperative recovery of hip joint function [13]. The EQ-5D was used to evaluate living quality [14]. Nonunion was defined as persistence of the fracture line 6 months after the surgical procedure [15]. Femoral head necrosis was judged according to the criteria described by Slobogean et al. [16]. Femoral neck shortening was identified by measuring the difference in the length of on the affected side compared with the normal side on pelvic orthotopic X-ray photographs [17]. Fixation failure was defined as fracture displacement (> 5 mm) and femoral neck shortening (> 10 mm), nail withdrawal, or varus deformity (> 10°) compared with initial reduction [18].

### Statistical analysis

Continuous data were expressed as mean ± standard deviation (SD) and analyzed using the independent-samples *t* test. Categorical data were expressed as absolute numbers or percentages and analyzed using χ^2^ test. Statistical analyses were performed using SPSS 19.0 software (SPSS Inc, USA). A *P* value < 0.05 was considered statistically different.

## Results

### Baseline characteristics of subjects in the combination vs control groups

Finally, 156 subjects (102 males and 54 females) were selected in our study, and they all completed visits during the follow-up, as shown in Fig. 1. Of all subjects, 76 received allograft fibular cannulated screw fixation and 80 accepted ordinary cannulated screw fixation, with a mean age of 40.3 ± 5.30 and 41.0 ± 6.30 years for the two groups (Table 1). There were no statistically significant differences in age, gender, the mechanism of injury, the side of fracture, time to surgery (3.77 ± 0.82 vs. 3.64 ± 1.0 days, *P* = 0.383), BMI (23.5 ± 2.58 vs. 24.1 ± 2.50, *P* = 0.17), Garden classification, Pauwels classification and follow-up duration (11.3 ± 1.42 vs. 10.9 ± 1.72 years, *P* = 0.207) between the combination and control groups (*P* > 0.05). The baseline clinical and demographic characteristics of these subjects are summarized in Table 1.

**Table 1:** Comparison of baseline characteristics between the combination versus control groups

Items	Groups		*P*-value
	Combination group (*n* = 76)	Control group (*n* = 80)	
Age (years)	40.3 ± 5.30	41.0 ± 6.30	0.458
Gender (%)			
Male	52(68.4)	50(62.5)	0.473
Female	24(31.6)	30(37.5)	
Mechanism of injury (%)			
High-energy trauma	51(67.1)	54(67.5)	0.850
Low-energy trauma	25(32.9)	26(32.5)	
Side of fracture			
Left	30(39.5)	33(41.3)	0.821
Right	46(60.5)	47(58.7)	
Time to surgery (days)	3.77 ± 0.82	3.64 ± 1.0	0.383
BMI	23.5 ± 2.58	24.1 ± 2.50	0.17
Garden classification			
I	0	0	0.997
II	15	16	
III	33	35	
IV	28	29	
Pauwels classification			
I	2	3	0.721
II	40	46	
III	34	31	
Follow-up duration (years)	11.3 ± 1.42	10.9 ± 1.72	0.207

### Operative time, blood loss, and hospital stay in the combination vs control groups

The combination group showed significantly longer mean operative time than the control group (61.0 ± 5.46 vs. 55.9 ± 5.67 min, *P* < 0.001). This suggested that fibula allograft combined with cannulated screw fixation slightly prolonged the operation time. There was more mean intra-operative blood loss in the combination group than that in the control group, with a significant difference between the two groups (66.4 ± 5.61 vs. 63.6 ± 8.03 ml, *P* = 0.013). No remarkable difference in the length of hospital stay was found between the two groups (6.80 ± 1.83 vs. 6.51 ± 1.49, *P* = 0.209) (Table 2).

**Table 2:** Comparison of operative time, blood loss, hospital stay between the combination *v.s.* control groups

Items	Groups		*P*-value
	Combination group (*n* = 76)	Control group (*n* = 80)	
Operative time (min)	61.0 ± 5.46	55.9 ± 5.67	< 0.001
Intra-operative blood loss (ml)	66.4 ± 5.61	63.6 ± 8.03	0.013
Length of hospital stay (day)	6.80 ± 1.38	6.51 ± 1.49	0.209

### Healing time, full weight-bearing stepping, HHS and EQ-5D scores in the two groups

The mean healing time of femoral neck fractures in the combination group was significantly shorter than that in the control group (3.5 ± 0.84 vs. 3.86 ± 0.91 months, *P* = 0.016), indicating a superiority of the combination strategy in functional recovery. The time course of recovery of full weight-bearing was significantly shorter in the combination group than that in the control group (3.6 ± 0.39 vs. 4.1 ± 0.5 months, *P* < 0.001). As for postoperative hip function of the two groups, there was a nonsignificant difference in the HHS score between the two groups at 3-month (60.4 ± 7.10 vs. 59.0 ± 6.36, *P* = 0.613) and 1-year (80.8 ± 7.15 vs. 79.3 ± 6.99, *P* = 0.195) follow-up. Strikingly, the HHS scores at 3-year and 8-year follow-up in the combination group (90.2 ± 2.86 vs. 91.8 ± 4.85) were significantly higher than those in the control group (85.4 ± 5.49 vs. 86.8 ± 6.62), respectively (*P* < 0.001). With respect to postoperative quality of life, the two groups showed similar EQ-5D scores at 3-month (0.41 ± 0.079 vs. 0.38 ± 0.087, *P* = 0.061) and 1-year follow-up (0.72 ± 0.053 vs. 0.70 ± 0.062, *P* > 0.153). The combination group exhibited significantly higher EQ-5D scores at 3-year (0.83 ± 0.47 vs. 0.81 ± 0.59, *P* = 0.005) and 8-year (0.85 ± 0.50 vs. 0.82 ± 0.07, *P* = 0.003) follow-up than the control group (Table 3).

**Table 3:** Comparison of healing time, full weight-bearing time, HHS score and EQ-5D between the combination versus control groups

Items	Groups		*P*-value
	Combination group (*n* = 76)	Control group (*n* = 80)	
Healing time (months)	3.5 ± 0.84	3.86 ± 0.91	0.016
Time course of recovery of full weight-bearing (months)	3.6 ± 0.39	4.1 ± 0.52	< 0.001
HSS			
3-month	60.4 ± 7.10	59.0 ± 6.36	0.613
1-year	80.8 ± 7.15	79.3 ± 6.99	0.195
3-year	90.2 ± 2.86	85.4 ± 5.49	< 0.001
8-year	91.8 ± 4.85	86.8 ± 6.62	< 0.001
EQ-5D			
3-month	0.41 ± 0.079	0.38 ± 0.087	0.061
1-year	0.72 ± 0.053	0.70 ± 0.062	0.153
3-year	0.83 ± 0.47	0.81 ± 0.59	0.005
8-year	0.85 ± 0.50	0.82 ± 0.07	0.003

### Postoperative and bone healing complications in the combination vs control group

**Fig. 5:**
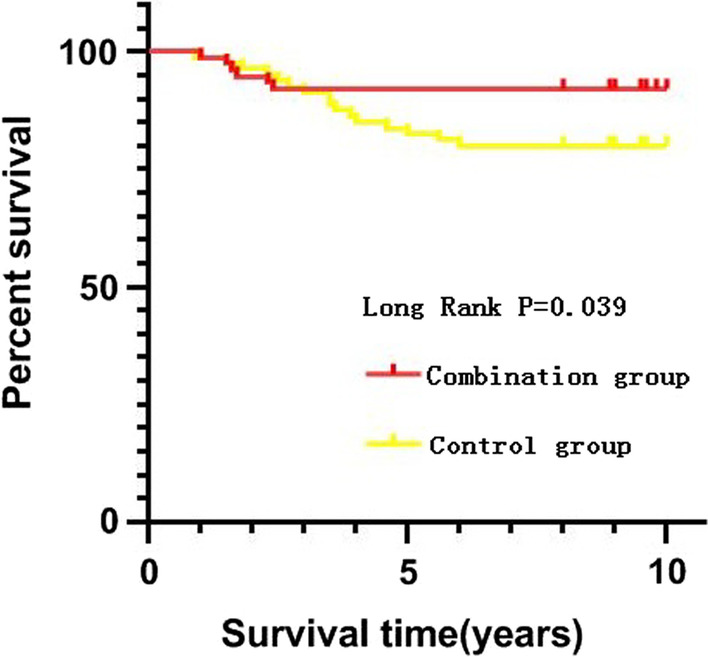
Kaplan–Meier survival analysis for the survival rate of the femoral head of the two groups. The 10-year survival rate of the femoral head in combination group was significantly higher than that in the control group

The rate of femoral head necrosis was significantly lower in the combination group than that in the control group (30.3% vs. 37.5%, *P* = 0.049). That table shows that a high percentage of patients in the control group progressed to femoral osteonecrosis in 37.5%, although 63.5% of patients eventually healed (Table 4). In addition, the rate of fracture nonunion significantly decreased in the combination group compared with the control group (2.6% vs. 11.3%, *P* = 0.036). However, there was no statistical difference in the rate of fixation failure between the two groups (*P* = 0.336). The rates of femoral neck shortening > 5 mm in the horizontal (*x*) and vertical (*y*) were higher in the control group than that in the combination group (*x*: 20.0% vs. 6.6%, *P* = 0.014; *y*: 17.5% vs. 5.3%, *P* = 0.017). About 6.6% subjects receiving fibula allograft with cannulated screw fixation for femoral neck fracture underwent total hip replacement surgery, which was significantly lower than the proportion of 17.5% in the control group (*P* = 0.031) (Table 4). The Kaplan–Meier survival analysis was performed to assess the survival rate of the femoral head, with the total hip replacement as the end point. The survival rate was compared between the two groups using the Mantel-Cox log-rank test. As shown in Fig. 5, the 10-year survival rate of the femoral head in the combination group was significantly higher than that in the control group (93.4% *vs* 80%, *P* = 0.039).

**Table 4:** Comparison of postoperative and bone healing complications between the combination *v.s.* control groups

Variables	Groups		*P*-value
	Combination group (*n* = 76)	Control group (*n* = 80)	
Femoral head necrosis (%)	23(30.3)	30(37.5)	0.049
Nonunion (%)	2(2.6)	9(11.3)	0.036
Failure of fixation (%)	1(1.3)	3(3.75)	0.336
Femoral neck shortening (> 5 mm)			
Horizontal-plane (%)	5(6.6)	16(20)	0.014
Vertical-plane (%)	4(5.3)	14(17.5)	0.017
Total hip replacement (%)	5(6.6)	16(20)	0.031

## Discussion

This study analyzed and compared the long-term clinical outcomes and complications of 156 femoral neck fracture patients receiving fibula allograft combined with cannulated screw fixation versus ordinary cannulated screw fixation. All subjects were followed up during an average of 11.1 years and were eligible for this study. The results showed that there were better performances in healing time, the time course of recovery of full-weight-bearing stepping, HHS and EQ-5D scores in the combination group than those in the control group. The rates of femoral head necrosis, nonunion, femoral neck shortening and total hip replacement were significantly lower in the combination group than those in the control group.

With the incidence of high-energy injuries (e.g., traffic injuries and falling injuries) increasing, the prevalence of femoral neck fractures is rising in young and middle-aged people [19]. Due to the special anatomy of the femoral neck, the incidence of postoperative complications encompassing nonunion and femoral head necrosis are still high, which seriously affects hip joint functional recovery and the quality of life of these patients [6]. Therefore, improving functional recovery and reducing complications after internal fixation surgery have been the hot spot and the bottleneck. Ordinary cannulated screw fixation is a common treatment for femoral neck fractures with the advantages of less invasive, less intra-operative blood loss, shorter hospital stay, and operative time [20, 21]. However, as for Pauwels type III femoral neck fractures that are relatively unstable with a high risk of postoperative complications and high rates of mechanical failure after internal fixation, dynamic hip screws are considered to have lower rates of necrosis and nonunion compared to ordinary cannulated screw fixation [20, 22, 23]. Torsional tests in biomechanics found that the torsional stiffness and maximum torque of the dynamic hip screw were greater than those of the cannulated screw, and its maximum torque was less than that of the cannulated screw, indicating that the dynamic hip screw is more biomechanically stable [24]. Nonetheless, more complicated operation procedures and longer surgery time of dynamic hip screw determine more blood loss intraoperatively. In current clinical practice, there is no optimal surgical stabilization method for femoral neck fractures [7].

Some studies indicated that concomitant bone grafting and screw fixation is the option for displaced fractures, as well as can minimize the probability of necrosis. Baksi et al. [25] reported bone grafts with muscle pedicles for patients with nonunion of femoral neck fractures, and the rate of fracture healing was up to 82%. Regrettably, the surgical procedures are complicated, time-consuming, and invasive. Roshan et al. [26] reported 32 cases of old femoral neck fractures treated with two cannulated screw fixation combined with regional block in free fibula flaps, and no femoral head necrosis occurred. Our long-term results showed a shorter healing time and recovery time course to full weight bearing in the combined group than in the control group, improving a faster postoperative recovery in patients who received allograft fibula grafts combined with cannulated screw fixation. Despite nonsignificant differences in HHS and EQ-5D scores between the two groups within 1-year follow-up, the scores at 3- and 8-year follow-up were higher in the combination group than those in the control group, indicating that fibula allograft with cannulated screw fixation improves patients' long-term hip joint function and the quality of life. Although there were differences in the statistical results of HHS and EQ-5D between the two groups, their actual clinical scores were relatively close. The reason for differences could be that in the early postoperative period, two groups of patients needed time to recover from surgical trauma, blood loss, and other aspects of body damage. Furthermore, individual differences in subjective perception of pain or function led to slight differences in HHS and EQ-5D scores between the two groups, which remained statistically insignificant. As for the follow-up from 3 to 8 years after surgery, HHS scores in the combined treatment group were higher than in the hollow screw-only group due to the greater relief of pain and other symptoms after a longer recovery period. Different from the HHS scores, the EQ-5D system places more emphasis on subjective feelings, which may account for the small differences in EQ-5D scores between the two groups.

Additionally, compared with ordinary cannulated screw fixation, patients receiving fibula allograft with cannulated screw fixation reported lower incidence rates of complications such as femoral head necrosis (30.3%), nonunion (2.6%), femoral neck shortening (6.6%). The incidence of total hip replacement (6.6%) was also reduced compared with the control group. Stability can create a good biomechanical environment for fracture healing and repair of femoral head necrosis. It is noteworthy that patients with femoral neck fractures of Garden types III and IV were included in this study in more than half of the total number of cases, with these subtypes usually being associated with higher rates of necrosis [27]. Therefore, the overall necrosis rate in this study was higher compared to other studies.

Our study did indicate that fibula allograft combined with cannulated screw fixation can achieve the initial stability and the long-term stability. Fibula allograft with cannulated screw fixation remarkably increases biomechanical stability to effectively prevent the femoral head from rotating and sinking during fracture reduction. Therefore, this combination strategy prominently promotes fracture healing as well as early recovery from fractures. In contrast to ordinary cannulated screw fixation, the contact area between the fibula and the subchondral bone plate of the femoral head is large enough, which thereby avoids stress concentration and minimizes a risk of penetrating the hip joint surface. More interesting, as the allograft fibula is hollow, it helps to reduce the pressure on the femoral head and reduce the incidence of necrosis. If femoral head necrosis occurs, the allogeneic fibula will provide a stable mechanical environment for repair of necrotic lesions.

The limitations of this study must be acknowledged. First, the number of cases is insufficient and no comparative study in different subgroups based on Garden and Pauwels classifications has been conducted. Second, the present study had an insufficient level of evidence as a retrospective study compared with a prospective study. Finally, as this study is a monocenter study, potential selection bias might reduce the applicability of the results.

## Conclusion

Taken together, the present study suggests that fibula allograft combined with cannulated screw fixation for femoral neck fractures achieves better long-term clinical outcomes with fewer postoperative complications than ordinary cannulated screw fixation. Notably, the 10-year survival rate of the femoral head has been improved after fibula allograft with cannulated screw fixation.

## Data Availability

The datasets used or analyzed during the current study are available from the corresponding author on reasonable request.

## Abbreviations

HHS: Harris hip score
EQ-5D: EuroQoL five-dimension questionnaire
CT: Computed tomography
MRI: Magnetic resonance imaging
